# Influence of Vitamin D Binding Protein on Accuracy of 25-Hydroxyvitamin D Measurement Using the ADVIA Centaur Vitamin D Total Assay

**DOI:** 10.1155/2014/691679

**Published:** 2014-06-19

**Authors:** James Freeman, Kimberly Wilson, Ryan Spears, Victoria Shalhoub, Paul Sibley

**Affiliations:** Siemens Healthcare Diagnostics, 511 Benedict Avenue, Tarrytown, NY 10591, USA

## Abstract

Vitamin D status in different populations relies on accurate measurement of total serum 25-hydroxyvitamin D [25(OH)D] concentrations [i.e., 25(OH)D_3_ and 25(OH)D_2_]. This study evaluated agreement between the ADVIA Centaur Vitamin D Total assay for 25(OH)D testing (traceable to the NIST-Ghent reference method procedure) and a liquid chromatography tandem mass spectrometry (LC-MS/MS) method for various populations with different levels of vitamin D binding protein (DBP). Total serum 25(OH)D concentrations were measured for 36 pregnant women, 40 hemodialysis patients, and 30 samples (DBP-spiked or not) from healthy subjects. ELISA measured DBP levels. The mean serum DBP concentrations were higher for pregnancy (415 *μ*g/mL) and lower for hemodialysis subjects (198 *μ*g/mL) than for healthy subjects and were highest for spiked serum (545 *μ*g/mL). The average bias between the ADVIA Centaur assay and the LC-MS/MS method was −1.4% (healthy), −6.1% (pregnancy), and 4.4% (hemodialysis). The slightly greater bias for samples from some pregnancy and hemodialysis subjects with serum DBP levels outside of the normal healthy range fell within a clinically acceptable range—reflected by analysis of their low-range (≤136 *μ*g/mL), medium-range (137–559 *μ*g/mL), and high-range (≥560 *μ*g/mL) DBP groups. Thus, the ADVIA Centaur Vitamin D Total assay demonstrates acceptable performance compared with an LC-MS/MS method for populations containing different amounts of DBP.

## 1. Introduction

Increasing awareness of the important role of vitamin D for bone and other diseases has led to increased 25-hydroxyvitamin D [25(OH)D] testing (D represents D_3_ and D_2_ forms). However, variability within and between methods and laboratories has often compromised correct diagnosis and the ability to compare results from different studies and national surveys [1–5]. Automated antibody-based, radioimmunoassays, high performance liquid chromatography (HPLC), and mass spectrometry methods for 25(OH)D testing are subject to variability issues that can arise from a variety of sources, such as differential detection of the D_3_ and D_2_ forms, interference by detection polyclonal antibodies, and nonspecific detection of other vitamin D metabolites such as the 3-epimer form of 25(OH)D [3-epi-25(OH)D] and 24,25(OH)_2_D_3_. In addition, incomplete release of 25(OH)D from the vitamin D binding protein (DBP) has been identified as a potential source of variability for both manual and automated immunoassays [6].

Establishing an immunoassay for 25(OH)D is challenging because the majority of the highly hydrophobic 25(OH)D is tightly bound (dissociation constant, Kd, 5 × 10^−8^ M) to a vast excess of DBP from which it must be separated; almost no 25(OH)D is found “free” (non-protein bound) in the circulation, and less than 5% of the available DBP binding sites are occupied by vitamin D compounds [7]. In addition, DBP binds vitamin D_3_ along with other metabolites and vitamin D_2_, whose similar structures may be easier to release from DBP and difficult to differentiate; DBP has a higher affinity for vitamin D_3_ than other metabolites and vitamin D_2_ [8]; and generating specific antibodies against small antigenic molecules, such as 25(OH)D, is difficult, but it is mandatory because the Vitamin D Standardization Program (VDSP) states that 25(OH)D assays should measure equimolar amounts of 25(OH)D_3_ and 25(OH)D_2_ (total vitamin D) [9]. Measuring total vitamin D is required because some supplements contain the D_2_ form, and not measuring D_2_ would lead to lower 25(OH)D values. In methods such as radioimmunoassay, HPLC, and mass spectrometry, an initial extraction step with organic solvents releases all bound 25(OH)D from DBP [10–13]. However, organic solvents are not compatible with most automated immunoassays, and alternative releasing agents, which are proprietary, are used instead. Recent studies performed in populations with different levels of DBP have questioned the effectiveness of these proprietary releasing agents to completely free 25(OH)D from DBP [6].

The goal of this study was to examine the ability of the ADVIA Centaur Vitamin D Total assay by comparison with an LC-MS/MS method to accurately measure 25(OH)D levels in serum samples from healthy adults (endogenous) and healthy adults with exogenous DBP (endogenous + spiked) and from pregnant women and chronic kidney disease (CKD) patients receiving dialysis, who have higher and lower than normal serum levels of DBP, respectively [7, 14, 15]. The ADVIA Centaur Vitamin D Total assay is traceable to the NIST-Ghent reference measurement procedure (RMP) for vitamin D testing. (This version of the ADVIA Centaur Vitamin D Total assay is not currently available commercially in all regions, including the USA.)

## 2. Materials and Methods

In order to determine the influence of DBP on a vitamin D immunoassay, a study examining DBP as an endogenous interference, similar to how hemoglobin, cholesterol, or total protein would be measured, following Clinical and Laboratory Standards Institute (CLSI) Document EP7-A2 [16] was performed at the Siemens R&D facility in Tarrytown, NY, USA. Human native DBP (>95% pure) was purchased from Athens Research & Technology, Inc.

### 2.1. LC-MS/MS

The LC-MS/MS method used in this study is traceable to the Esoterix ID-LC-MS/MS method, which is traceable to NIST. The LC-MS/MS method performed at Siemens used the Waters Acquity H-class ultrahigh performance liquid chromatography (UPLC) and triple quadrupole (TQD) tandem mass spectroscopy (MS) with MassLynx and QuanLynx software (Waters Acquity TQD system, Waters Corporation, Manchester, UK). This method is able to separate, identify, and separately quantify the concentrations of 25(OH)D_2_, 25(OH)D_3_, and 3-epi-25(OH)D_3_ in a serum sample. As reported by the manufacturer, the LC-MS/MS method demonstrated a dynamic assay range of 2.5–220 ng/mL (6.25–550 nmol/L) (*r* ^2^ > 0.997). Three levels of 25(OH)D_2_ and 25(OH)D_3_ concentrations tested over five consecutive days yielded intra-assay coefficients of variation (CVs) of ≤7.7% and interassay precision CVs of <12% for 25(OH)D_2_ and 25(OH)D_3_.

### 2.2. ADVIA Centaur Vitamin D Total Assay

The ADVIA Centaur Vitamin D Total assay used in this study is traceable to the Ghent University ID-LC-MS/MS 25(OH)D RMP. (This version of the assay is not currently available commercially in all regions, including the USA.) The Ghent University is a reference laboratory for the Vitamin D Standardization Program (VDSP). The sample reference material (SRM) used for the Ghent University method is traceable to the NIST SRM 2972 [4, 9, 17]. Recently, Siemens received confirmation from the Centers for Disease Control and Prevention (CDC) that the ADVIA Centaur Vitamin D Total assay is now a certified procedure of the Vitamin D Standardization-Certification Program (VDSCP). Certification was achieved by demonstrating that the total vitamin D [25(OH)D] results for the 40 VDSCP samples (10 quarterly challenges) agreed with the results from the ID-LC-MS/MS RMP method. The ADVIA Centaur Vitamin D Total assay achieved a mean bias of 0.3% (acceptance criterion was ±5.0%) and a mean imprecision of 5.5% (acceptance criterion was <10.0%). The ADVIA Centaur Vitamin D Total assay is a one-pass, 18-minute antibody competitive chemiluminescent immunoassay. Release of the 25(OH)D metabolites from the DBP is accomplished by denaturing and blocking agents. 25(OH)D in serum competes with a 25(OH)D analog (labeled with fluorescein) for an anti-25(OH)D monoclonal mouse antibody (labeled with acridinium ester). Detection occurs after the remaining anti-25(OH)D monoclonal antibody (labeled with acridinium ester) complexes with vitamin D analog (labeled with fluorescein) and anti-fluorescein monoclonal antibody covalently bound to paramagnetic particles. Results are inversely related to 25(OH)D serum concentrations. The standardized assay demonstrates equimolar cross-reactivity with 25(OH)D_2_ (104.5%) and 25(OH)D_3_ (100.7%), minimal cross-reactivity with 3-epimer-25(OH)D_3_ (1.1%), and a broad dynamic assay range of 4.2–150 ng/mL (10.5–375 nmol/L) [18]. Precision was determined by assaying six samples twice a day in replicates of 4, over 20 days (*n* = 160 replicates per sample) according to the Clinical and Laboratory Standards Institute (CLSI) protocol EP5-A2 [19]; the run-to-run CVs were in the range of 4.2% and 11.9% [18]. All samples were run in singlicate on both the LC-MS/MS and a single ADVIA Centaur system.

### 2.3. Sample Population

Clinical serum samples from 18 healthy adults were purchased from a commercial vendor (ProMedDx, Norton, MA, USA). Serum samples from 36 pregnant women in their third trimester and 40 CKD hemodialysis patients were purchased from another commercial vendor (Research Sample Bank, Delray Beach, FL, USA).

### 2.4. Samples

Peripheral venous blood samples were collected, placed at 4°C, and centrifuged; serum aliquots were prepared and stored for less than four months at –20°C until analysis. Generally, no difference was found in the serum concentrations of DBP for men and women [11, 15].

### 2.5. Protocol

Serum samples were sent to Siemens Healthcare Diagnostics (Tarrytown, NY, USA) for DBP and 25(OH)D measurements. The serum samples from the 18 healthy adults were divided into five serum pools; each of the four pools contained 4 individual serum samples and one pool contained 2 individual serum samples. The 25(OH)D concentrations in these five serum pools were measured by using a LC-MS/MS method at Siemens Healthcare Diagnostics, (Tarrytown, NY, USA) according to a protocol that allowed resolution of 25(OH)D_2_ and 25(OH)D_3_ from 3-epi-25(OH)D_3_. The LC-MS/MS values for the five individual pools (pools 1–5) resulted in mean 25(OH)D concentrations of 24, 32, 51, 41, and 75 ng/mL, respectively. The endogenous levels of DBP were measured in each of the five serum pools using the Quantikine ELISA Vitamin D Binding Protein BP kit, DVDBP0 (R&D Systems, Inc.). Subsequently, each of the five serum pools was divided into six aliquots, and DBP (ranging from 50 to 250 ug/mL in 50 ug/mL increments) was spiked into 5 of the 6 aliquots from each pool (Table 3). The DBP content in the resulting thirty samples was then reanalyzed to confirm higher DBP concentrations in spiked samples, and 25(OH)D measurements were performed according to routine procedures using the ADVIA Centaur Vitamin D Total assay traceable to the Ghent University ID-LC-MS/MS 25(OH)D RMP. (This version of the assay is not currently available commercially in all regions, including the USA.) Bias of 25(OH)D values to the original LC-MS/MS values was determined. In addition, the 36 clinical serum samples from third-trimester pregnancy patients and the 40 clinical serum samples from CKD patients were evaluated for endogenous DBP and 25(OH)D using the ADVIA Centaur Vitamin D Total assay; and bias of 25(OH)D values to the original LC-MS/MS values was determined. Only four samples from pregnancy subjects had detectable 25(OH)D_2_ (3.2, 5.2, 8.0, and 10.7 *μ*g/mL). Nineteen samples from dialysis patients had detectable 25(OH)D_2_ (range 1.6 to 35 *μ*g/mL), eight of which had levels above 10 *μ*g/mL. The 3-epi-25(OH)D_3_ was present at levels greater than 1.5 ng/mL in samples from 23 dialysis and 32 pregnancy subjects.

### 2.6. Statistics

Difference plots and bias values were obtained using Microsoft Excel (2010); Analyze-It add-in program in Excel was used to compare the different sets of data in order to obtain the 95% confidence interval (CI) and standard deviations (SD) for 95% limits of agreement. Correlation plots and correlation and Deming regression analyses were generated using GraphPad Prism, version 6.

## 3. Results 

The mean serum concentrations of DBP in healthy subjects (endogenous and endogenous + spiked), pregnant women, and dialysis patients are presented in Table 1. For the five serum pools, the average endogenous serum DBP concentration (±SD) was 348 ± 106 *μ*g/mL (range 260.7 to 519.0 *μ*g/mL), which is consistent with the results of other studies [6, 20, 21]. For healthy serum samples spiked with DBP, the average DBP concentration was higher (545 ± 185 *μ*g/mL, range 261.2 to 980.6 *μ*g/mL) than endogenous levels. For pregnancy samples, the average DBP concentration was also greater (415 ± 245 *μ*g/mL, range 82.2 to 874.5 *μ*g/mL) than that for healthy serum samples. In contrast, for CKD patients receiving dialysis, the average DBP concentration was lower (198 ± 173 *μ*g/mL, range 63.4 to 1115.7 *μ*g/mL; median 142.1 *μ*g/mL) than levels in healthy serum and pregnancy samples.

The mean total serum 25(OH)D concentrations and range as measured by the LC-MS/MS method and the ADVIA Centaur Vitamin D Total assay are presented in Table 2. The mean 25(OH)D levels (±SD) were 44.6 ± 19.8 and 44.8 ± 20.1 ng/mL for healthy serum samples (endogenous), 44.6 ± 18.0 and 43.5 ± 16.7 ng/mL for healthy (endogenous + spiked) serum samples, and 44.6 ± 18.0 and 43.7 ± 17.0 ng/mL for both endogenous and endogenous + spiked healthy serum samples, and they were lower for pregnancy serum samples, 27.3 ± 9.6 and 25.3 ± 8.7 ng/mL, and dialysis serum samples, 28.1 ± 14.8 and 29 ± 15.3 ng/mL. Consistent with previous reports, no correlation was found between the DBP and 25(OH)D concentrations for serum from dialysis patients (Pearson's correlation coefficient *r* = 0.1) [11, 14, 15]. Pregnancy samples demonstrated a positive correlation (*r* = 0.35; *P* = 0.013) between serum concentrations of DBP and 25(OH)D for LC-MS/MS, but no correlation was found for ADVIA Centaur (*r* = 0.15; *P* = 0.37). There were too few non-spiked healthy samples for valid 25(OH)D and DBP correlation assessment.

**Figure 1:**
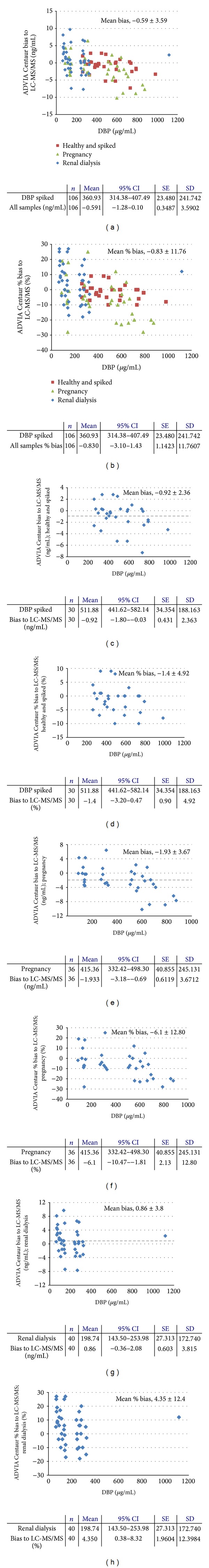
Bias and percent bias between the 25(OH)D results of the ADVIA Centaur Vitamin D Total assay and the LC-MS/MS method as a function of DBP concentration in healthy human serum pooled samples (endogenous and endogenous + spiked) (a, b, c, d), pregnancy (third trimester) samples (a, b, e, f), and renal dialysis samples (a, b, g, h). The bias ±1.96 standard deviation (SD) represents the 95% limits of agreement. To convert 25(OH)D concentrations to nanomoles per liter (nmol/L), multiply by 2.5.

The overall average bias of all samples from healthy individuals (endogenous and endogenous + spiked) for the ADVIA Centaur Vitamin Total assay to the LC-MS/MS method was –1.4%; for all third-trimester pregnancy samples, the average bias was –6.1%; and for all renal dialysis samples, the average bias was 4.4%. The results for bias, percent bias, 95% CI, and SD (95% limits of agreement = 1.96 SD) as a function of DBP concentration for each population—separate and combined—are presented in Figure 1. When all populations were combined, positive bias (versus LC-MS/MS) was observed at very low serum DBP concentrations and negative bias was observed at very high serum DBP concentrations (Figure 1).

**Figure 2:**
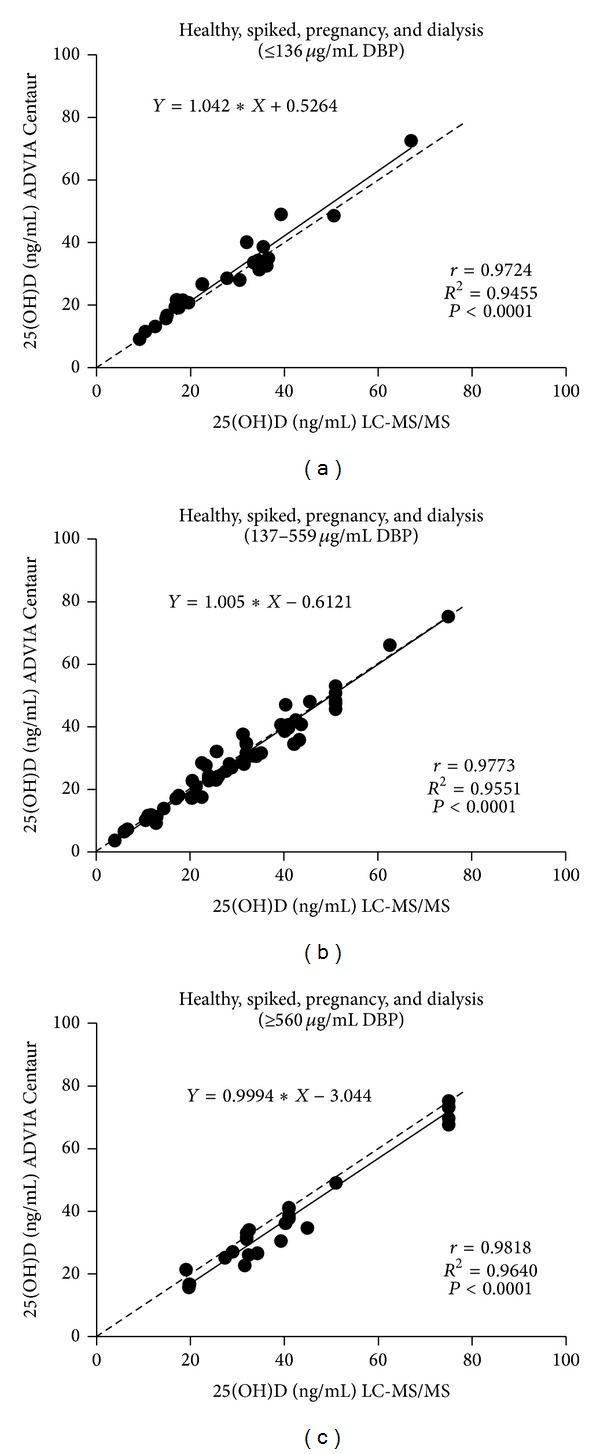
Correlation of 25(OH)D results obtained from the ADVIA Centaur Vitamin D Total assay and the LC-MS/MS method for combined normal human serum pooled samples (endogenous and endogenous + spiked), pregnancy (third trimester samples), and renal dialysis samples for (a) low, (b) medium, and (c) high DBP groups. Dotted line: line of identity. To convert 25(OH)D concentrations to nanomoles per liter (nmol/L), multiply by 2.5.

**Figure 3:**
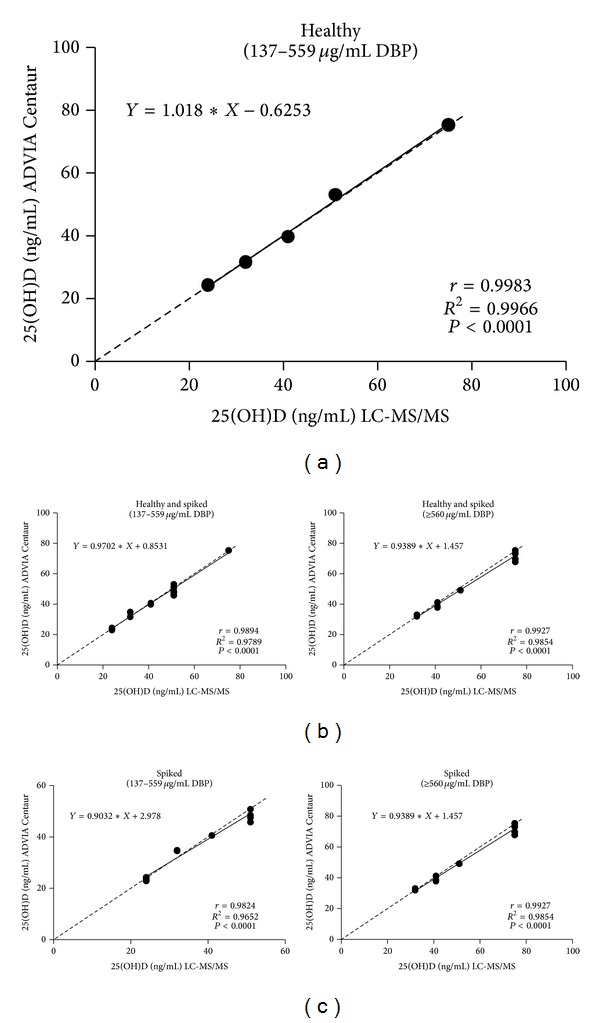
Correlation of 25(OH)D results obtained from the ADVIA Centaur Vitamin D Total assay and the LC-MS/MS method for normal human serum pooled samples: (a) endogenous, (b) endogenous and endogenous + spiked, and (c) endogenous + spiked. Dotted line: line of identity. To convert 25(OH)D concentrations to nanomoles per liter (nmol/L), multiply by 2.5.

**Figure 4:**
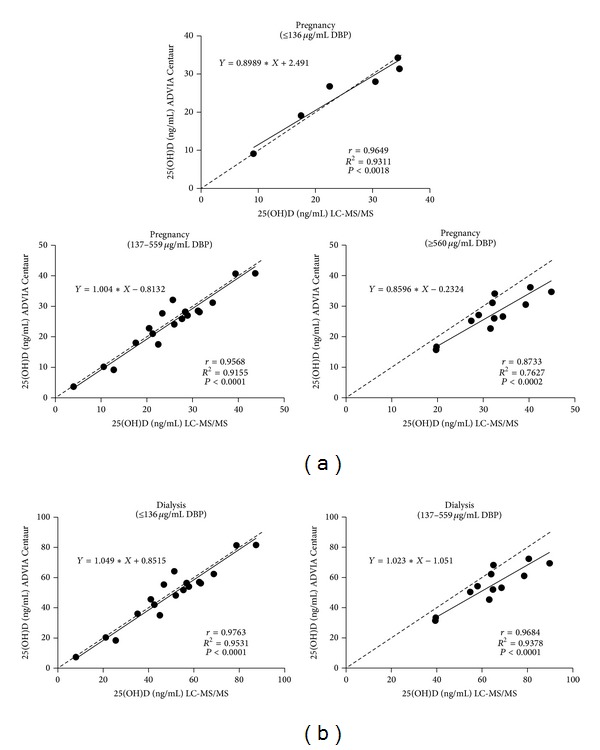
Correlation of 25(OH)D results obtained from the ADVIA Centaur Vitamin D Total assay and the LC-MS/MS method for (a) pregnancy (third trimester) samples and (b) renal dialysis samples. Dotted line: line of identity. To convert 25(OH)D concentrations to nanomoles per liter (nmol/L), multiply by 2.5.

With respect to dialysis samples with generally lower DBP concentrations, we do not know if uremic serum properties contributed to bias, and we question the validity of analyzing combined populations. Nevertheless, we examined how well the methods in subjects with serum DBP concentrations at extremes of the serum DBP concentration range (very low and very high)—for combined and separate populations (Figures 2, 3, and 4). Very low and very high serum DBP concentrations were defined as two SD below and above the mean for healthy subjects which is 348 ± 106 *μ*g/mL; hence, the very low DBP group comprised samples having concentrations of ≤136 *μ*g/mL, and high DBP group comprised samples having concentrations of ≥560 *μ*g/mL. The middle range group had samples with DBP concentrations ranging from 137 to 559 *μ*g/mL. The following populations were analyzed: (1) healthy, spiked, pregnancy, and dialysis (Figure 2); (2) healthy (which had no low or high groups) [Figure 3(a)]; (3) healthy and spiked (which had no low group) [Figure 3(b)]; (4) spiked (which had no low group) [Figure 3(c)]; (5) pregnancy [Figure 4(a)]; (6) dialysis (which had no high group) [Figure 4(b)].

Analysis of 25(OH)D values for ADVIA Centaur and LC-MS/MS as a function of low, medium, and high serum DBP concentrations demonstrated that correlations between the two methods were acceptable at low and high serum DBP levels for all populations analyzed (combined and separate) (Figures 2, 3, and 4), with pregnancy samples demonstrating the lowest correlation at very high serum DBP concentrations (*r* = 0.87, *P* < 0.0002). Healthy samples (endogenous and endogenous + spiked) showed very good correlations and agreement between methods. The mean bias obtained for combined populations and each population separately for their low, medium, and high range DBP groups represented acceptable assay performance (Table 4).

LC-MS/MS identified 25(OH)D_3_, 25(OH)D_2_, and 3-epi-25(OH)D_3_ in samples. Only four samples from pregnant subjects had detectable 25(OH)D_2_ (3.2, 5.2, 8.0, and 10.7 *μ*g/mL). Nineteen samples from dialysis patients had detectable 25(OH)D_2_ (range 1.6 to 35 ng/mL), eight of which had levels above 10 ng/mL. The mean percent bias of the eight 25(OH)D_2_ samples which had greater than 10 ng/mL was 9.0 ± 0.12% (mean ± SD), whereas the mean percent bias of the remaining samples was 3.0 ± 0.12%. Of the 40 dialysis patients, 17 had less than 1.5 ng/mL 3-epi-25(OH)D_3_ and 23 (58%) had 3-epi-25(OH)D_3_ concentrations ranging from 1.7 to 3.6 (mean ± SD, 2.5 ± 0.57 ng/mL). Of the 36 pregnancy subjects, four had less than 1.5 ng/mL 3-epi-25(OH)D_3_ and 32 (89%) had 3-epi-25(OH)D_3_ concentrations ranging from 1.6 to 6.3 ng/mL (mean ± SD, 3.3 ± 1.3).

## 4. Discussion

This study addressed the influence of DBP on the accuracy of the ADVIA Centaur Vitamin D Total assay by comparison with an LC-MS/MS method. The ADVIA Centaur Vitamin D Total assay results in this study are traceable to ID-LC-MS/MS 25(OH)D reference method procedure and the standard reference materials established by NIST and the University of Ghent [4, 9, 17, 22].

In healthy individuals, endogenous serum DBP concentration (347.6 *μ*g/mL) was found to be within the range reported by others (300–600 *μ*g/mL) [20] and increased after DBP spiking (545 *μ*g/mL). Despite the increase in DBP concentrations after spiking, 25(OH)D measurements in individual samples were equivalent between the two methods. There were no healthy (endogenous or endogenous + spiked) samples in the low DBP range, as defined by two SD values below the mean of healthy samples (i.e., ≤136 *μ*g/mL) (Figure 3). The high DBP range, as defined by two SD values above the mean of healthy samples (i.e., ≥560 *μ*g/mL), comprised twelve spiked samples (Figure 3), and 25(OH)D agreement was good between the methods for 25(OH)D values (*r* = 0.9927, *P* < 0.0001; bias −3.0 ± 3.98%). Thus, there was not a significant bias observed for the ADVIA Centaur for healthy samples (endogenous + spiked). This demonstrates that DBP concentrations as high as 980 *μ*g/mL did not appear to interfere with the assay for this population. By comparison, endogenous serum DBP concentrations peaked at 519 *μ*g/mL in healthy subjects. Because the use of DBP-spiked samples may be suspect, these results will be confirmed in future studies which evaluate a greater number of samples from healthy subjects containing endogenous serum DBP concentrations in the higher range (although it is unlikely that normal healthy subjects exist with DBP concentrations that can be achieved at the high spiking concentrations).

Depending on hormonal status or disease state serum matrix components may be different, and the levels of DBP may be higher or lower than those of healthy individuals [14, 15]. In women who are receiving estrogen therapy and those who are pregnant, higher serum estrogen levels correlate with increases in circulating DBP and total 1,25(OH)_2_D. During pregnancy, increased 1,25(OH)_2_D_3_ occurs in response to the growing calcium demands of the fetus [14, 15]. Consistent with these reports, the mean DBP concentration was greater (415 *μ*g/mL) for samples from pregnant women than for those from healthy subjects (347.6 *μ*g/mL) and dialysis patients (198 *μ*g/mL). Despite the overall higher DBP concentrations in pregnancy serum, 25(OH)D results for those samples with low, medium, and high DBP concentrations demonstrated acceptable agreement between the ADVIA Centaur Vitamin D Total assay and the LC-MS/MS method (*r* = 0.96, *P* < 0.0018, bias 2.0 ± 10.9%; *r* = 0.96, *P* < 0.0001, bias –3.0 ± 12.6%; *r* = 0.87, *P* < 0.0002, bias –14.0 ± 9.9%, resp.). Although samples in the low and medium DBP range showed less bias than those with very high DBP concentrations, the assay performance was acceptable for all groups. Four samples out of 36 contained measureable 25(OH)D_2_; it is unlikely that 25(OH)D_2_ influenced the assay bias because several samples lacking 25(OH)D_2_ demonstrated similar levels of bias. In contrast to a previous study that found higher 25(OH)D levels in pregnant women compared to nonpregnant healthy women, this study found overall lower levels in pregnant women; this difference may relate to differences in vitamin D supplementation [6].

Nephrotic syndrome and CKD predialysis and dialysis patients demonstrate diminished serum levels of the bioactive 1,25(OH)_2_D, likely due, in part, to impaired renal synthesis, nutritional deficit, and lower 25(OH)D substrate levels [23–26]. Although some studies report no change in serum DBP levels in renal failure patients compared with healthy individuals, other studies demonstrate lower serum levels and increased DBP urinary excretion; lower serum concentrations of DBP likely reflect increased urinary loss due to proteinuria, which is a common finding in CKD patients [15, 27, 28]. In this study, the overall mean 25(OH)D level was equivalent between the ADVIA Centaur Vitamin D Total assay and the LC-MS/MS method and for the low and medium range DBP groups (*r* = 0.97 overall; *r* = 0.98 low range DBP group; *r* = 0.97 middle range DBP group, *P* < 0.0001; bias was 4.35 ± 12.4% overall, 10.0 ± 10.6% for the low range DBP group and 0.0 ± 12.4% for the middle range DBP group, resp.), indicating acceptable performance of the ADVIA Centaur Vitamin D Total assay in the presence of DBP and uremic serum. It is not known whether unique components of uremic serum contributed to the bias observed. Nineteen samples from dialysis patients had detectable 25(OH)D_2_ (range 1.6 to 35 ng/mL), eight of which had levels above 10 ng/mL. The 25(OH)D_2_ containing samples appeared to contribute to the positive bias in this patient population. This result is consistent with the performance of the ADVIA Centaur Vitamin D Total assay which demonstrates a slight difference in recovery for 25(OH)D_2_ and 25(OH)D_3_ (104.5% versus 100.7%) as stated in the Instructions for Use Manual [18]. Only one uremic sample was found in the higher range [1115.7 *μ*g/mL DBP; 19.1 ng/mL 25(OH)D_3_ by LC-MS/MS and 21.4 ng/mL 25(OH)D by ADVIA Centaur; 12% bias of ADVIA Centaur to LC-MS/MS]. Whether an error in DBP measurement was the cause for the unusually high DBP concentration is not known. Although the serum 25(OH)D concentrations in dialysis patients were lower than those found in healthy individuals, the values were approximately normal (according to the Endocrine Society Guidelines). This is likely due to patient adherence to vitamin D supplementation which is indicated for end-stage renal disease patients on dialysis. It is worth noting that lower levels of serum 25(OH)D concentrations in predialysis patients correlate with a greater risk of mortality [29]. This underscores the need to accurately evaluate and monitor serum 25(OH)D levels in the CKD patient population.

A recent study implicated ineffective 25(OH)D-DBP extraction procedures as the cause of variability in an evaluation of five automated assays compared to an RMP LC-MS/MS method [6]. The study, which included samples from healthy individuals, pregnant women, dialysis patients, and intensive care patients, found that the bias was, at least in part, dependent on DBP concentration. The ADVIA Centaur Vitamin D Total assay in the present study differed from the assay in the previous study in that it has a different standardization; this version is standardized with internal standards traceable to the NIST-Ghent VDSP RMP. This may have had some impact on why the results of this study differ from those previously reported.

## 5. Conclusions

The small positive bias found in renal dialysis patients with DBP concentrations below those found in normal healthy subjects and small negative bias found in pregnant subjects with DBP levels above those found in normal healthy subjects were within the acceptable range for the assay. Thus, for populations with different levels of DBP, the 25(OH)D results obtained by the ADVIA Centaur Vitamin D Total immunoassay were equivalent to the sum of 25(OH)D_2_ and 25(OH)D_3_ using the LC-MS/MS method—especially for individuals with serum DBP concentrations within the range for the healthy population (137 to 559 *μ*g/mL).

## Figures and Tables

**Table 1:** Serum concentrations of vitamin D binding protein in healthy subjects, DBP-spiked samples from healthy subjects, pregnant women, and dialysis patients.

	Number of samples	Average ± SD (*μ*g/mL)	Range (*μ*g/mL)	Median (*μ*g/mL)	Interquartile (IQ) range (*μ*g/mL)
Healthy not spiked (endogenous)	5	348 ± 106	261–519	ND	ND
Healthy (endogenous and endogenous + spiked)	30	512 ± 188^a^	261–981	ND	ND
Healthy (endogenous + spiked)	25	545 ± 185^a^	261–981	ND	ND
Pregnancy	36	415 ± 245^a^	82–875	515	150–599
Dialysis	40	198 ± 173	63–1116	142	100–262

^a^ *P* < 0.0001 compared to the dialysis group.

ND: not determined.

DBP: vitamin D binding protein.

**Table 2:** Serum concentrations of 25(OH)D in healthy subjects, DBP-spiked samples from healthy subjects, pregnant women, and dialysis patients.

	Number of samples	ADVIA Centaur Vitamin D Total assay	ADVIA Centaur Vitamin D Total assay	LC-MS/MS	LC-MS/MS
		Average ± SD (ng/mL)	Range (ng/mL)	Average ± SD (ng/mL)	Range (ng/mL)
Healthy not spiked (endogenous)	5	44.8 ± 20.1^a^	24.0–75.0	44.6 ± 19.8	24.3–75.3
Healthy (endogenous and endogenous + spiked)	30	43.7 ± 16.7^c,d^	22.9–75.3	44.6 ± 18.0^e^	24.0–75.0
Healthy (endogenous + spiked)	25	43.5 ± 16.7^b,d^	22.9–75.3	44.6 ± 18.0^e^	24.0–75.0
Pregnancy	36	25.3 ± 8.7	3.7–40.8	27.3 ± 9.6	4.0–44.9
Dialysis	40	29.0 ± 15.3	6.5–72.6	28.1 ± 14.8	6.0–67.0

To convert 25(OH)D concentrations to nanomoles per liter (nmol/L), multiply by 2.5.

^a^ *P* < 0.05 compared to the pregnancy group; ^b^ *P* < 0.01 compared to the dialysis group; ^c^ *P* < 0.001 compared to the dialysis group; ^d^ *P* < 0.0001 compared to the pregnancy group; ^e^ *P* < 0.001 compared to the pregnancy and dialysis groups.

DBP: vitamin D binding protein.

**Table 3:** Serum concentrations of DBP in healthy subjects and DBP-spiked samples from healthy subjects.

LC-MS/MS (ng/mL)	Concentration of spiked DBP in serum (*μ*g/mL)	DBP (mg/mL)	ADVIA Centaur (ng/mL)	ADVIA Centaur bias to LC-MS/MS
24		276.9	24.3	1%
24	50	347.2	24.2	1%
24	100	385.5	23.5	−2%
24	150	334.9	24.3	1%
24	200	407.1	23.1	−4%
24	250	472.1	22.9	−5%

32		301.6	31.6	−1%
32	50	339.1	34.8	9%
32	100	629.7	33	3%
32	150	446.8	34.8	9%
32	200	489.5	34.5	8%
32	250	584.4	31.9	0%

51		260.7	53	4%
51	50	261.2	48.4	−5%
51	100	327.8	47.5	−7%
51	150	417.7	50.8	0%
51	200	593.3	49.1	−4%
51	250	486.2	45.7	−10%

41		380.1	39.7	−3%
41	50	420.5	40.6	−1%
41	100	590.2	41.2	0%
41	150	747.3	38.5	−6%
41	200	738	40.9	0%
41	250	980.6	37.7	−8%

75		519	75.3	0%
75	50	584.8	69.7	−7%
75	100	724.9	75.3	0%
75	150	788.4	73.4	−2%
75	200	731.3	67.7	−10%
75	250	789.7	73.1	−2%

DBP: vitamin D binding protein. To convert 25(OH)D concentrations to nanomoles per liter (nmol/L), multiply by 2.5.

**Table 4:** Mean bias (±SD) compared to LC-MS/MS for the low, medium, and high range DBP groups for combined populations and each population separately: healthy and DBP-spiked, DBP-spiked, pregnant women, and dialysis patients.

	DBP ≤136 *μ*g/mL	DBP 137–559 *μ*g/mL	DBP ≥560 *μ*g/mL
Bias (ng/mL)			
Combined populations	1.67 ± 3.33 (*n* = 24)	−0.45 ± 3.07 (*n* = 57)	−3.0 ± 3.98 (*n* = 25)
Healthy (endogenous and endogenous + spiked)		−2.78 ± 2.12 (*n* = 18)	−1.88 ± 2.47 (*n* = 12)
Healthy (endogenous + spiked)		−0.45 ± 2.40 (*n* = 13)	−1.88 ± 2.47 (*n* = 12)
Pregnancy	0.017 ± 2.76 *n* = 6	−0.72 ± 0.13 (*n* = 18)	−4.72 ± 3.67 (*n* = 12)
Dialysis	2.23 ± 3.38 (*n* = 18)	−0.38 ± 3.91 (*n* = 21)	2.30 (*n* = 1)
% bias			
Combined populations	8.0 ± 10.99% (*n* = 24)	−1.0 ± 10.66% (*n* = 57)	−8.0 ± 10.09% (*n* = 25)
Healthy (endogenous and endogenous + spiked)		0.0 ± 5.30% (*n* = 18)	−3.0 ± 3.98% (*n* = 12)
Healthy (endogenous + spiked)		0.0 ± 6.10% (*n* = 13)	−3.0% ± 3.98% (*n* = 12)
Pregnancy	2.0 ± 10.9% (*n* = 6)	−3.0 ± 12.6% (*n* = 18)	−14.0 ± 9.9% (*n* = 12)
Dialysis	10.0 ± 10.6% (*n* = 18)	0.0 ± 12.4% (*n* = 21)	12.0% (*n* = 1)

DBP: vitamin D binding protein. To convert 25(OH)D concentrations to nanomoles per liter (nmol/L), multiply by 2.5.

## References

[1] Carter GD (2011). Accuracy of 25-hydroxyvitamin D assays: confronting the issues. *Current Drug Targets*.

[2] Carter GD, Berry JL, Gunter E (2010). Proficiency testing of 25-hydroxyvitamin D (25-OHD) assays. *Journal of Steroid Biochemistry and Molecular Biology*.

[3] Hollis BW (2004). Editorial: the determination of circulating 25-hydroxyvitamin D: no easy task. *Journal of Clinical Endocrinology and Metabolism*.

[4] Sempos CT, Vesper HW, Phinney KW, Thienpont LM, Coates PM, Vitamin D Standardization Program (VDSP) (2012). Vitamin D status as an international issue: national surveys and the problem of standardization. *Scandinavian Journal of Clinical and Laboratory Investigation*.

[5] Wallace AM, Gibson S, de la Hunty A, Lamberg-Allardt C, Ashwell M (2010). Measurement of 25-hydroxyvitamin D in the clinical laboratory: current procedures, performance characteristics and limitations. *Steroids*.

[6] Heijboer AC, Blankenstein MA, Kema IP, Buijs MM (2012). Accuracy of 6 routine 25-hydroxyvitamin D assays: influence of vitamin D binding protein concentration. *Clinical Chemistry*.

[7] Cooke NE, Haddad JG (1989). Vitamin D binding protein (Gc-globulin). *Endocrine Reviews*.

[8] Hollis BW (1984). Comparison of equilibrium and disequilibrium assay conditions for ergocalciferol, cholecalciferol and their major metabolites. *Journal of Steroid Biochemistry*.

[9] Thienpont LM, Stepman HCM, Vesper HW (2012). Standardization of measurements of 25-Hydroxyvitamin D3 and D2. *Scandinavian Journal of Clinical and Laboratory Investigation*.

[10] US Renal Data System (2007). *Annual Data Report: Atlas of End-Stage Renal Disease in the United States: International Comparisons*.

[11] Bouillon R, van Baelen H, de Moor P (1977). The measurement of the vitamin D binding protein in human serum. *Journal of Clinical Endocrinology and Metabolism*.

[12] Gilbertson TJ, Stryd RP (1977). High-performance liquid chromatographic assay for 25-hydroxyvitamin D3 in serum. *Clinical Chemistry*.

[13] Justova V, Starka L, Wilczek H, Pacovsky V (1976). A simple radioassay for 25 hydroxycholecalciferol without chromatography. *Clinica Chimica Acta*.

[14] Bouillon R, van Assche FA, van Baelen H, Heyns W, de Moor P (1981). Influence of the vitamin D-binding protein on the serum concentration of 1,25-dihydroxyvitamin D3. Significance of the free 1,25-dihydroxyvitamin D3 concentration. *Journal of Clinical Investigation*.

[15] Haddad JG, Walgate J (1976). Radioimmunoassay of the binding protein for vitamin D and its metabolites in human serum. Concentrations in normal subjects and patients with disorders of mineral homeostasis. *Journal of Clinical Investigation*.

[16] Clinical and Laboratory Standards Institute (NCCLS) (2005). *Interference Testing in Clinical Chemistry*.

[17] Stepman HCM, Vanderroost A, van Uytfanghe K, Thienpont LM (2011). Candidate reference measurement procedures for serum 25-hydroxyvitamin D3and 25-hydroxyvitamin D2 by using isotope-dilution liquid chromatography-tandem mass spectrometry. *Clinical Chemistry*.

[18] (2013). *Siemens ADVIA Centaur XP Vitamin D Total (VitD) Assay*.

[19] Clinical and Laboratory Standards Institute (NCCLS) (2004). *Evaluation of Precision Performance of Quantitative Measurement Methods*.

[20] Kawakami M, Blum CB, Ramakrishnan R, Dell RB, Goodman DS (1981). Turnover of the plasma bindng protein for vitamin D and its metabolites in normal human subjects. *Journal of Clinical Endocrinology and Metabolism*.

[21] Speeckaert M, Huang G, Delanghe JR, Taes YEC (2006). Biological and clinical aspects of the vitamin D binding protein (Gc-globulin) and its polymorphism. *Clinica Chimica Acta*.

[22] Miller WG, Myers GL, Gantzer ML (2011). Roadmap for harmonization of clinical laboratory measurement procedures. *Clinical Chemistry*.

[23] Al-Badr W, Martin KJ (2008). Vitamin D and kidney disease. *Clinical Journal of the American Society of Nephrology*.

[24] González EA, Sachdeva A, Oliver DA, Martin KJ (2004). Vitamin D insufficiency and deficiency in chronic kidney disease: a single center observational study. *The American Journal of Nephrology*.

[25] LaClair RE, Hellman RN, Karp SL (2005). Prevalence of calcidiol deficiency in CKD: a cross-sectional study across latitudes in the United States. *The American Journal of Kidney Diseases*.

[26] Williams S, Malatesta K, Norris K (2009). Vitamin D and chronic kidney disease. *Ethnicity & Disease*.

[27] Thrailkill KM, Jo C, Cockrell GE, Moreau CS, Fowlkes JL (2011). Enhanced excretion of vitamin D binding protein in type 1 diabetes: a role in vitamin D deficiency?. *Journal of Clinical Endocrinology and Metabolism*.

[28] van Hoof HJC, de Sévaux RGL, van Baelen H (2001). Relationship between free and total 1,25-dihydroxyvitamin D in conditions of modified binding. *European Journal of Endocrinology*.

[29] Mehrotra R, Kermah DA, Salusky IB (2009). Chronic kidney disease, hypovitaminosis D, and mortality in the United States. *Kidney International*.

